# Identification of serum predictors of n-acetyl-l-cysteine and isoproterenol induced remodelling in cardiac hypertrophy

**DOI:** 10.3906/biy-2101-56

**Published:** 2021-06-23

**Authors:** Dharaniyambigai KUBERAPANDIAN, Victor Arokia DOSS

**Affiliations:** 1 Department of Biochemistry, PSG College of Arts & Science, Coimbatore, Tamil Nadu India

**Keywords:** Cardiac hypertrophy, serum predictors, reductive stress, metabolic remodeling, n-acetyl-L-cysteine, isoproterenol

## Abstract

Cardiac hypertrophy (CH), leading to cardiac failure is due to chronic metabolic alterations occurring during cellular stress. Besides the already known relationship between oxidative stress and CH, there are implications of reductive stress leading to CH. This study attempted to develop reductive stress-based CH rat model using n-acetyl-L-cysteine (NAC), a glutathione agonist that was compared with typical isoproterenol (ISO) induced CH model. The main objective was to identify serum metabolites that can serve as potent predictors for seven routine clinical and diagnostic parameters in CH: 3-hydroxybutyrate (3-HB), lactic acid (LA), urea, and ECG-CH parameters (QRS complex, R-amplitude, R-R interval, heart rate) that were hypothesized to underlie metabolic remodelling in this study. CH was assessed using electrocardiography, hypertrophic index and histopathological analysis (H&E stain) in both ventricles after 2 weeks. Gas chromatography mass spectroscopy analysis (GC-MS) identified unique metabolite finger-prints. Correlation and pattern analysis revealed strong relationships between specific metabolites and parameters (Pearson’s score > 0.7) of this study. Multiple regression analysis (MRA) for the strongly related metabolites (independent variables) with each of the seven parameters (dependent variables) identified significant predictors for the latter namely fructose, valine, butanoic acid in NAC and cholesterol, erythrose, isoleucine in ISO models, with proline and succinic acid as common for both models. Metabolite set enrichment analysis (MSEA) of those significant predictors (p < 0.05) mapped butyrate metabolism as highly influential pathway in NAC, with arginine-proline metabolism and branched chain amino acid (BCAA) degradation as common pathways in both models, thus providing new insights towards initial metabolic remodeling in the pathogenesis of CH.

## 1. Introduction

Cardiac hypertrophy (CH) is the gradual and asymptomatic enlargement and inflammation of ventricular walls characterized by accumulation of extracellular matrix (ECM) proteins. This accumulation was addressed as cardiac fibrosis (CF) which leads to contractile dysfunction and heart failure. CH is caused primarily by the metabolic alterations that are initiated as adaptive mechanisms for energy production during cellular stress related with metabolic diseases/syndromes like diabetes, hypertension, obesity, pregnancy, exposure to drug or radiotherapy, and vigorous exercises (Gibb and Hill, 2017).

Isoproterenol is a β-adrenergic receptor agonist which is used to develop CH model based on oxidative stress and impaired antioxidant systems through downregulated nuclear erythroid like factor 2 (Nrf2). Therapies that activate Nrf2, antioxidants, and reduced glutathione have been reported to ameliorate oxidative stress associated CH (Erkens et al., 2015). Reductive stress (RS) refers to the increase in the synthesis of reduced glutathione and NADH by endogenous mechanisms such as constitutively active and unregulated Nrf2-antioxidants, hyperglycemia, and vigorous exercise (Rajasekaran et al., 2005; Yan 2014).

The objectives of this study are to fundamentally understand the early remodelling events during reduction (RS) - oxidation (OS) referred as redox stress on the basis of metabolite shifts by developing in-vivo rat model of RS using n-acetyl-L-cysteine (NAC) and compared with the standard oxidative stress-based CH model, isoproterenol (ISO). The main objective of this study is the identification of predictors for seven routinely preferred clinical parameters in CH comorbidities namely 3-hydroxybutyrate (3-HB), lactic acid (LA), urea, and ECG peaks for CH (QRS complex, R-amplitude, R-R interval, heart rate) that were hypothesized to underlie metabolic remodelling in this study with the perspective of fore-casting CH through redox stress based metabolite remodelling. 

## 2. Materials and methods

### 2.1. Chemicals 

All chemicals and reagents used were analytical grade (Hi Media Pvt Ltd., India). N-trimethylsilyl-N-methyl trifluoroacetamide (MSTFA), methoxyamine hydrochloride, n-acetyl-L-cysteine, and isoproterenol (isoprenaline hydrochloride) were purchased from Sigma-Aldrich.

### 2.2. Experimental rats

Male Sprague Dawley rats (5 weeks old; 100 to 110 g body weight) procured after ethical clearance (CPCSEA/No.: 399/2018/IAEC) were acclimatized (3 days) under controlled temperature (29 ^o^C ± 5 ^o^C), humidity (55% ± 5%), and 12 h of light/dark cycles. They were divided into two groups (n = 5 rats each) and induced cardiac hypertrophy (CH) in 2 weeks: -

Group 1 – normal (control -NOR)

Group 2 – n-acetyl L-cysteine (NAC -1g/kg/day, oral) (Liu et al., 2015)

Group 3 - isoproterenol (ISO -10 mg/kg, i.p) (Doss et al., 2019)

### 2.3. Electrocardiography (ECG) analysis of CH 

In order to monitor the cardiac functions in vivo after 2 weeks, electrocardiography (ECG) was performed for 6 min at the conventional bipolar limb lead II using BITalino ECG Sensor – Open Signals[r]evolution software in unanesthetized rats. The changes in QRS complex, R amplitude, R–R interval, and pulse/heart beat rate (HR) were recorded. These changes were calculated as: [(ECG parameter value in CH model - in NOR)/(in NOR) x100] (Konopelski and Ufnal, 2016; Doss and Kuberapandian, 2019).

### 2.4. Hypertrophy index 

The heart size in a rat model was determined by the heart weight to body weight ratio (Doss and Kuberapandian, 2019).

### 2.5. Histopathological analysis 

The heart tissues were excised and initially preserved in 10% formaldehyde until their left and right ventricles were processed and stained with haematoxylin and eosin (H&E). Cellular architecture was examined at 40x magnification (Zahkouk et al., 2015; Doss et al., 2019)

### 2.6. Gas chromatography-mass spectroscopy analysis

Serum was isolated from the cardiac blood (punctured after over-night fasting) as per the modified protocol (Sowndarya and Doss, 2017). Briefly, serum (100 µL) was precipitated using acetonitrile (250 µL), evaporated to dryness (N_2_ gas), and treated with methoxylamine hydrochloride (20 mg/mL in pyridine) at 70 °C for 60 min and MSTFA at 40 °C for 90 min. One µL of the sample was injected into the inlet port (10:1 split mode) and analysed using a Shimadzu GC-2010 plus gas chromatography instrument coupled to a Shimadzu QP2010 mass spectrometer (Shimadzu, Japan) with helium (carrier gas) at flow rate of 1 mL/min with following conditions: initial temperature (100 °C for 4 min) to 270 °C (5 °C/min); injection (280 °C); interface (250 °C); ion source (200 °C); solvent delay (9 min). MS was operated in electron ionisation mode (70 eV; m/z of 35 to 800) followed by the identification of metabolites based on Nist & Wiley mass library (ribitol as reference).

### 2.7. Statistical analysis

Specific and comprehensive analysis using Pearson’s correlation and pattern analysis along with metabolite set enrichment analysis (MSEA - SMPDB library) were done using MetaboAnalyst 4.0 (Chong et al., 2019). Multiple regression analysis and individual linear regression prediction models were performed using Statistical Package for Social Sciences v: 26.0 (SPSS IBM, Armonk, NY, USA). This software verified data which was expressed as mean ± standard error by one way analysis of variance (ANOVA) with significance set at p < 0.05 (Sowndarya and Doss, 2017; Dhakal, 2019; Doss and Kuberapandian, 2019).

## 3. Results

### 3.1. ECG screening for CH in DC

Widened QRS complex with prolonged R-R interval and impaired HR were observed in both the ISO and NAC models when compared to normal. The NAC administered rats displayed shortened R amplitude, unlike the standard CH model, ISO group that revealed elevated R-amplitude (Table 1 and Figure S1). 

**Table 1:** Screening CH characteristics and ventricular function using ECG.

Groups	QRS complex (ms)	% change	R amplitude (mV)	% change	R-R interval (ms)	% change	Heart rate (HR) (bpm)	% change
I - NOR	19.30 ± 0.52	-	0.43 ± 0.05	-	162.49 ± 2.93	-	369.00 ± 1.77	-
II - NAC	16.70 ± 0.10a	13.86	0.30 ± 0.08 a*	30.90	180.00 ± 5.98a*	10.73	334.00 ± 10.93 a*	9.58
III - ISO	21.00 ± 4.51b	9.11	0.80 ± 0.07 b*	87.85	198.00 ± 2.55 b*	21.85	303.00 ± 3.90 b*	17.95

Table 1 values the mean ± S.E of 5 samples per group (ms – millisecond; bpm – beats per minute). The percentage changes in each ECG parameters are indicated by red (increase) and blue (decrease) arrows. Group comparison: a – NOR vs. NAC; b – NOR vs. ISO. Statistical significance (p < 0.05) indicated by *.

### 3.2 Hypertrophy index

Morphologically, enlarged ventricles were observed in both NAC and ISO models when compared to the normal model (Figure S2). Significant decrease in body weight was observed in NAC model when compared to the normal and ISO models. Though, the heart weight was decreased in NAC which was contrary to the standard ISO model, the heart weight to body weight ratio was significantly increased in the NAC model (Table 2).

**Table 2:** Hypertrophic indices among experimental rats.

Groups	Body weight(BW in g)	Heart weight (HW in g)	HW/BW ratio
I - NOR	110.36 ± 3.65	0.320 ± 0.010	2.92 ± 0.12
II - NAC	40.00 ± 3.65 a*	0.363 ± 0.005 a*	9.05 ± 0.95 a*
III - ISO	90.00 ± 7.30 b*	0.750 ± 19.58 b*	8.41 ± 0.71 b*

Table 2 shows the mean ± S.E of 5 samples per group. Group comparison: a – NOR vs. NAC; b – NOR vs. ISO. Statistical significance (p < 0.05) indicated by*.

### 3.3. Histopathological analysis 

When compared to the normal and ISO groups, NAC administration revealed thickened and distorted myofibrillar architecture of the left and right ventricles (Figure S3). 

### 3.4. Gas chromatography-mass spectroscopy analysis 

The shifts among 26 metabolites along with 3-hydroxybutyrate, lactic acid, and urea were revealed in the sera of NAC and ISO when compared to normal. On comparison with the normal serum, glutamic acid, 2-deoxygalactose, and succinic acid were found at high levels in ISO and NAC samples with very low levels of serum cholesterol. Butanoic acid was exclusively high in NAC that also indicated only trace levels of 3-hydroxybutyrate. Similarly, mannonic acid, ribose and erythrose were observed intensely in ISO group whereas galactose and mannose were found at trace levels (Table 3).

**Table 3:** Concentration of serum metabolites and unique fingerprint metabolites in experimental groups identified using GC-MS analysis.

Metabolites (mmol mL–1)	Normal (NOR)	N-acetyl-L-cysteine (NAC)	Isoproterenol (ISO)
Amino acids Valine	1.60 ± 0.03	9.86 ± 0.22 a*	3.65 ± 0.15b
Leucine	2.51 ± 0.20	12.82 ± 0.21 a*	7.19 ± 0.11 b
Isoleucine	0.96 ± 0.09	0.03 ± 0.001 a*	4.08 ± 0.13 b
Serine	0.39 ± 0.05	17.89 ± 0.18 a*	7.81 ± 0.21 b*
Threonine	0.21 ± 0.14	19.26 ± 0.25 a*	9.27 ± 0.15 b*
Alanine	1.45 ± 0.26	1.91 ± 0.19 a	5.06 ± 0.13 b*
Glutamic acid	0.03 ± 0.001	19.32 ± 0.22 a*	11.40 ± 0.11 b*
Glycine	0.12 ± 0.03	3.55 ± 0.15 a	8.43 ± 0.23 b*
Proline	1.08 ± 0.11	2.80 ± 0.10 a*	3.40 ± 0.13 b*
Norleucine	0.01 ± 0.002	0.015 ± 0.0001 a*	1.38 ± 0.16 b*
Sugars Xylitol	1.48 ± 0.17	100.68 ± 0.02 a*	82.42 ± 0.16 b*
Fructose	1.29 ± 0.08	0.01 ± 0.002 a*	20.64 ± 0.23 b*
Galactose	13.84 ± 0.02	1.40 ± 0.08 a	0.08 ± 0.001 b*
Ribose	0.07 ± 0.003	0.01 ± 0.002 a*	47.19 ± 0.15 b*
Glucose	12.80 ± 0.08	182.72 ± 0.29 a*	160.50 ± 0.23 b*
Mannose	0.19 ± 0.007	8.31 ± 0.14 a*	0.03 ± 0.003 b*
Inositol	3.30 ± 0.04	21.17 ± 0.14 a*	22.78 ± 0.17 b*
Erythrose	0.09 ± 0.001	0.016 ± 0.0005 a*	1.54 ± 0.19 b*
Mannonic acid	0.04 ± 0.003	0.01 ± 0.001 a*	1.93 ± 0.12 b*
2-deoxy-D-galactopyranose	0.06 ± 0.002	3.52 ± 0.26 a*	3.51 ± 0.18 b*
Succinic acid	0.01 ± 0.002	3.78 ± 0.29 a	2.23 ± 0.16 b
Lipids Propanoic acid	0.66 ± 0.05	114.33 ± 0.61 a*	305.57 ± 0.24 b
Butanoic acid	0.06 ± 0.004	3.06 ± 0.23 a*	0.01 ± 0.001 b*
Palmitic acid	4.22 ± 0.05	29.93 ± 0.20 a*	39.68 ± 0.21 b*
Oleic acid	0.27 ± 0.01	7.27 ± 0.18 a	19.25 ± 0.27 b*
Cholesterol	1.63 ± 0.04	0.01 ± 0.004 a*	0.09 ± 0.003 b*
3-Hydroxybutyrate	3.60 ± 0.02	0.01 ± 0.002 a*	29.05 ± 0.26 b*
Lactic acid	12.85 ± 0.21	127.07 ± 0.27 a*	112.51 ± 0.25 b*
Urea	11.02 ± 0.07	379.44 ± 1.11 a*	97.57 ± 0.17 b

### 3.5. Correlation analysis 

**Figure 1:**
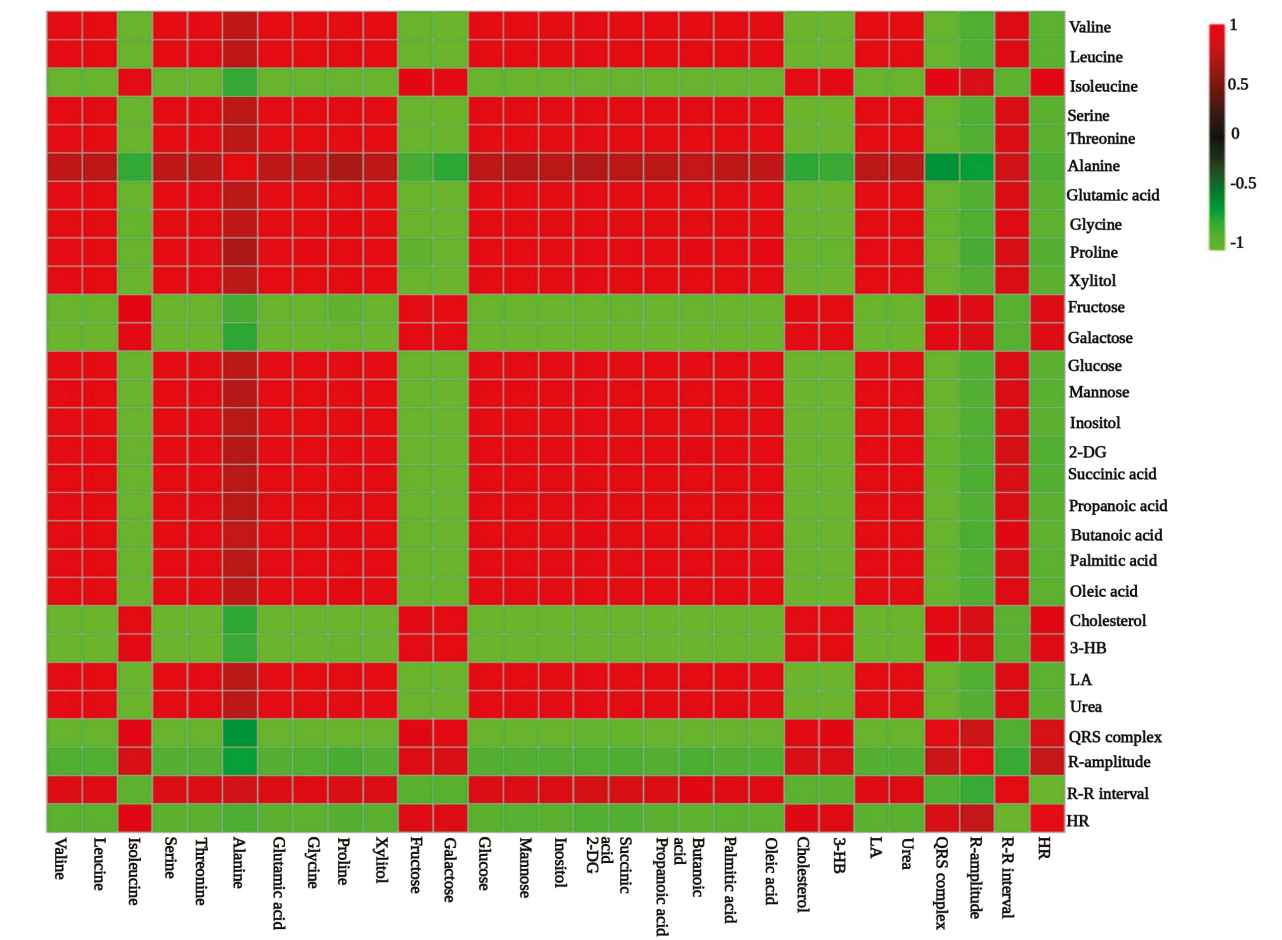
Correlation heat map for NAC induced CH. Pearson’s correlation analysis revealed strong correlation (scores ≥ 0.7) between most of the metabolites and with the seven metabolic responders in most of the serum metabolites in NAC model, in which alanine was observed as the least strong.

Pearson’s correlation analysis revealed the most strongly correlating metabolites between other metabolites and with the seven metabolic responders (3-hydroxybutyrate, lactic acid, urea, QRS complex, R-amplitude, R-R interval, heart rate) (Figure 1). Alanine was the least strong metabolite in the NAC model and no metabolites correlated with QRS complex in the ISO model. Isoleucine, fructose, galactose, and cholesterol correlated directly and strongly with heart rate in the NAC model, besides 3-hydroxybutyrate, QRS complex and R-amplitude. Galactose, mannose, and cholesterol correlated negatively and significantly with other metabolites in the ISO model (Figure S4). Correlation analysis accompanied by pattern hunting revealed the correlates for seven metabolic responders (Figure S5 and Figure S6).

### 3.6. Multiple regression analysis 

No significant predictors for R-amplitude in the NAC model and for QRS complex in the ISO model were depicted. Succinic acid and proline were the significant predictors commonly distributed in both CH models. The order of strong fit models with higher and significant R^2^ values were fructose > valine > proline > succinic acid > butanoic acid in NAC and proline > cholesterol > succinic acid > erythrose > isoleucine in the ISO model (Table 4 and Table 5).

**Table 4:** Predictors for metabolic responders in NAC induced CH models.

Metabolic responders	Potent predictor metabolite	Prediction equation	Predictor - linear model fitness and its significance
QRS complex	Proline	y = 23.596 + 0.971x	R2 = 1.00; p = 0.001*
R-amplitude	Valine	y = –0.159 + 0.113x	R2 = 0.54; p = 0.067NS
R-R interval	Butanoic acid	y = 185.418 + 15.537x	R2 = 0.77; p = 0.002*
Heart rate/pulse rate (HR)	Succinic acid	y = 310.825 + 28.664x	R2 = 0.97; p = 0.003*
3-HB	Fructose	y = 2.422 + 1.348x	R2 = 1.00; p = 8.2003E-7*
LA	Fructose	y = 134.113 + 9.851x	R2 =1.00; p = 0.00001*
Urea	Valine	y = 134.113 + 9.851x	R2 =1.00; p = 4.5625E-8*

Table 4 indicates the panel of metabolites (independent variable) that are potent predictor and their respective responder (dependent variable) for NAC induced CH model along with their model fitness wherein statistical significance (p < 0.05) indicated by * and nonsignificant as NS.

**Table 5:** Predictors for metabolic responders in ISO induced CH models.

Metabolic responders	Potent predictor metabolite	Prediction equation	Predictor - linear model fitness and its significance
QRS complex	---	---	---
R-amplitude	Erythrose	y = –0.310 + 0.407x	R2 = 0.95; p = 0.009*
R-R interval	Succinic acid	y = 225.208 + 15.339x	R2 = 0.97; p = 0.0002*
Heart rate/pulse rate (HR)	Cholesterol	y = 226.292 + 80.412x	R2 = 0.98; p = 0.001*
3-HB	Isoleucine	y = 32.334 + 0.598x	R2 = 0.73; p = 0.000007*
LA	Proline	y = 137.183 + 1.566x	R2 = 0.99; p = 1.9931E-8*
Urea	Proline	y = 103.677 + 2.956x	R2 = 1.00; p = 3.6906E-8*

### 3.7. Pathway mapping in the models

**Figure 2:**
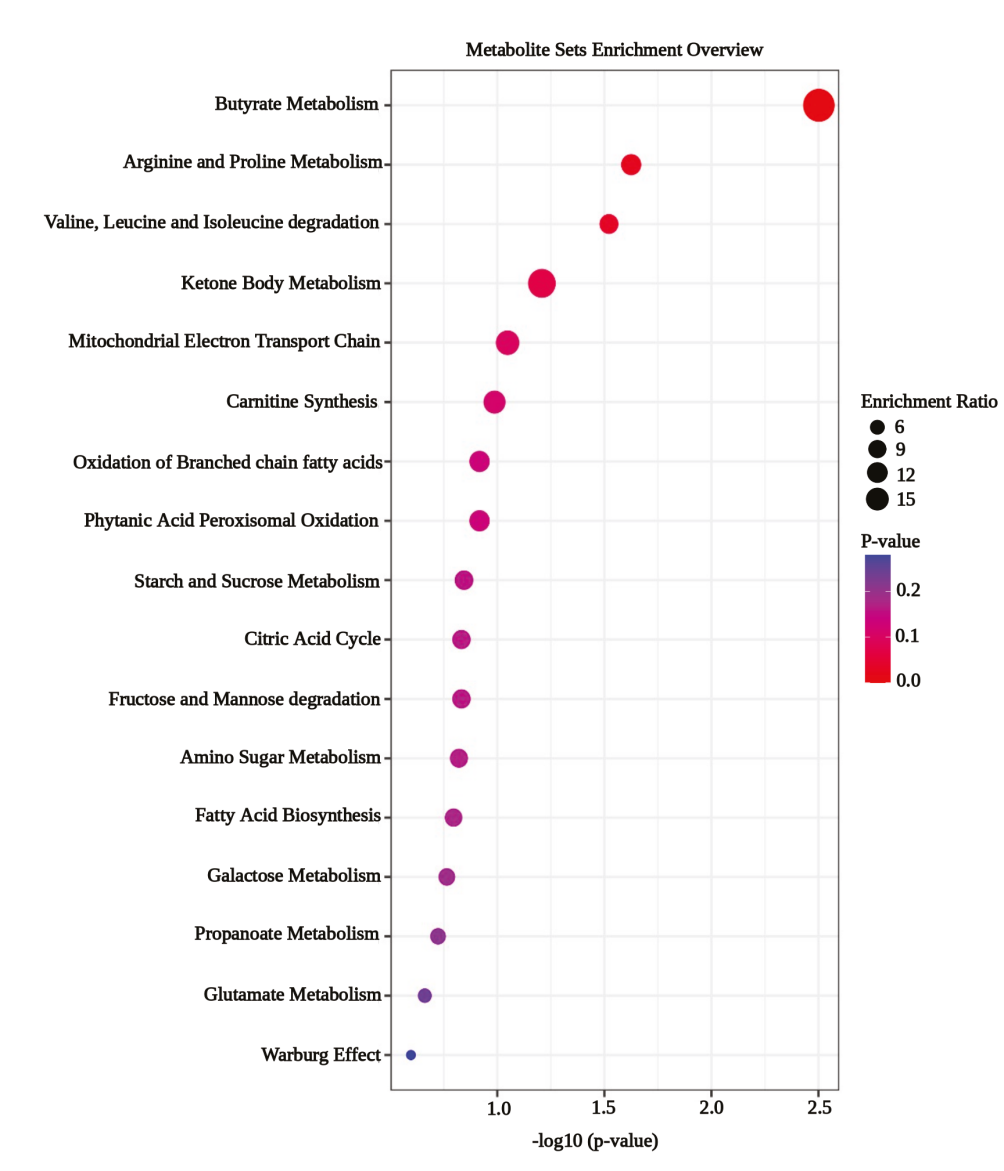
Representation of highly influenced pathways mapped using MSEA. Butyrate metabolism mapped as highly impacted pathway in NAC model followed by arginine and proline metabolism along with BCAA degradation that were also found to be common in ISO (see Figure S7).

Arginine and proline metabolism along with branched chain amino acid degradation were mapped as highly influenced common pathways in both the NAC and ISO models by MSEA. Butyrate metabolism was identified as the most significant pathway in the NAC model (p < 0.05). Moreover, analysis revealed the panel of other significantly influenced metabolic pathways but with less significance (Figure 2 and Figure S7).

## 4. Discussion

The primary indicator of CH are the typical electrocardiographic features such as widened QRS complex and prolonged R-R interval with delayed heart rate which were displayed in the ISO model of this study. On the other hand, the NAC model displayed unique characteristics such as shortened R-amplitude and prolonged R-R interval (Figure S1 and Table 1). Such a pattern has been previously reported linked to dilated cardiomyopathy, a condition that is closely associated with CH (Finocchiaro et al., 2020). This was further justified in this study by the elevated heart weight to body weight ratio in the NAC model when compared to the ISO and normal groups (Table 2 and Figure S2). Such an elevation in the heart weight to body weight ratio was attributed to extensive fibrosis (Wang et al., 2019). 

Histopathological analysis revealed substantially impaired cardiac architecture in NAC and ISO models when compared to the normal model. Although NAC had been previously reported as a potent antioxidant, few alternative studies report on the contrary (Liu et al., 2015). Thereby, NAC may trigger disease progression probably through induction of reductive stress through glutathione dependent mechanisms (Zhang et al., 2012). In this study, administration of NAC for 2 weeks could have induced accumulation of collagens and extracellular matrix proteins which have led to cardiac fibrosis. These changes were indicated by the decrease in R-amplitude, reduction in body weight (cachexia), and increase in hypertrophy index due to shift in metabolism towards energy sustenance (Edwards, 1974; Larssen et al., 2017). Though previously NAC was reported contrarily towards intervening cachexia in HIV patients, the same study indicated for a precaution about dosages (Droge et al.,1997; Delafontaine and Akao, 2006) as established in this study primarily through ECG and HW/BW ratio. This hereby warrants focussed pathophysiological understanding in terms of dilation and cachexia underlying the NAC and reductive stress in CH.

The respective list of predictors identified in NAC (reductive stress) and ISO (oxidative stress) models of CH in this study mapped branched chain amino acid (BCAA) degradation pathway as one of the mechanistic routes underlying pathological remodelling. Previous studies indicated the two possible sides of impaired branched chain amino acid catabolism: (i) increased leucine, valine, and isoleucine in CH due to low expression of catabolic enzymes and that excessive circulating leucine can influence metabolic homeostasis that can induce systolic dysfunction (Gibb and Hill, 2017) (ii) elevated plasma leucine can decrease the plasma levels of valine and isoleucine by affecting branched-chain ketoacid dehydrogenase (Tom and Nair, 2006). This study displays both of these phenomena as leucine levels were increased in both CH models, valine and isoleucine levels were decreased in the NAC model but they were raised in the ISO model (Table 3). The catabolites of isoleucine are ketogenic compounds namely acetoacetate, acetyl coenzyme and the glucogenic counterpart, succinyl coenzyme A (Tom and Nair, 2006). These elements of the ketone body metabolism could be the reason for isoleucine to be identified as a predictor of 3-hydroxybutyrate(3-HB) in the ISO model. Similarly, valine was identified as a predictor of urea in the NAC model of this study. Previous studies reported elevated valine levels as a biomarker of diabetic cardiomyopathy. In renal disorder associated with CH, urea production was associated with the amino acid catabolism that was influenced by valine levels (Veeneman et al., 2004; Liao et al., 2019). 

This study highlights proline as a crucial metabolite in CH. Proline is a scavenger of free radicals which are associated with glutathione biosynthesis, and this amino acid is exogenously used to overcome ventricular remodelling during oxidative stress (Wang et al., 2020). Contrarily, this study displayed elevated proline levels at the initial stages of CH in both the NAC and ISO models (Table 3) which could be due to hyperprolinemia type I (Wang et al., 2020) besides the participation of this elevated proline in collagen formation and fibrosis. Proline was also identified as a predictor for QRS complex in the NAC model besides its action upon lactic acid and urea in the ISO model (Table 4) which were substantially supported by the metabolite set enrichment analysis (Figure 2 and Figure S7). Previous studies indicated arginine as the source for proline and urea production since proline can decrease lactate dehydrogenase that produces lactate in the pathogenesis of CH (Revis and Cameron, 1978; Zheng et al., 2018; Wang et al.,2020). These could be the rationale for proline being identified as a predictor in the NAC and ISO models. 

In Figure 3, purple highlights are associated with the NAC model and brown highlights indicate the ISO model (diagram created using BioRenderBioRender.com [accessed on 06.06.2021]). Proline and succinic acid are common predictors identified in both CH models similar to the common BCAA and arginine-proline metabolisms. In this study, succinate was significantly elevated in both CH models when compared to the normal model. Previous researches have observed similar elevation in succinate levels during ischemia which may cause arrhythmias (Aguiar et al., 2014; Tretter et al., 2016). This might be the underlying mechanism for the reduction in R-R intervals and heart rates of the NAC and ISO models (Figure S1 and Table S1). This study hypothesizes that succinic acid may become a putative biomarker for CH and related dysrhythmia as it has been previously reported in association with ROS homeostasis besides its contribution towards energy metabolism. 

Previous studies reported about the reduced expression of biomarkers for CH in specific rat models through inhibition of histone deacetylases by sodium butyrate (Subramanian et al., 2016) wherein the beneficial effects of butyrate on cellular growth were also specified (Lui et al., 2018). In this study, butyrate metabolism was mapped as a pathway that can be impacted highly by the predictors of NAC (Figure 2). Butyric acid was significantly elevated in the NAC model and significantly lowered in the ISO model when compared to the normal model (Table 3). This finding shows both effects of butyrate related with the cellular stress mechanisms in the NAC and ISO models.

Another observation in this study was the low levels of fructose in the NAC model and elevated levels of fructose in the ISO model (Table 3). Previous studies reported that high levels of fructose are associated with oxidative stress, increased lactic acid production, and decreased ketone body production in the ISO model. The NAC model displaying very low levels of fructose could be due to aberrant fructose metabolism as a concomitant event of hypoxia inducible factor that is involved in the pathogenesis of various diseases including CH (Mirtschink et al., 2015). Thus, this study demonstrates the role of fructose metabolism which varies in NAC and ISO models. Fructose was identified as a predictor of lactic acid and 3-hydroxybutyrate (3-HB) in the NAC model (Table 4) which suggested that fructose levels could correlate with 3-hydroxy butyrate and lactic acid directly. This finding might explain the predictor relationship of fructose with its responders, 3-hydroxybutyrate and lactic acid in the NAC model (Rawat and Menahan, 1975; Yan 2014). 

Cholesterol levels in both CH models were significantly decreased (Table 4) which is in contrast to the well-established fact that hypercholesterolemia is associated with CH by affecting membrane cholesterol and impairing membrane receptors and ionic channels/pumps, inducing oxidative stress via NADP(H) oxidase. Hypocholesterolemia (low cholesterol) has been addressed as a cellular response for pathophysiological mechanisms of CH such as inflammation and in chronic heart failure (Horwich et al., 2002; Rauchhaus et al., 2003). These apparent mechanisms linked to arrhythmias hereby justifies the relationship of cholesterol with heart rate as the predictor and responder in the ISO model (Liao, 2004; Goonasekara et al., 2010) and thus warranting focus on hypocholesterolemia in CH besides hypercholesterolemia.

This study indicates the elevation in erythrose level in the ISO model and its identification as a predictor of R-amplitude. There is scarce data about the role of erythrose in energy metabolism and synthesis of aromatic amino acids such as phenylalanine and tryptophan during the pathogenesis of CH (Caggiano et al., 2020). 

The present study emphasizes the identification and relationship of eight significant metabolites as potent predictors for the seven routinely diagnosed clinical responders of CH comorbidities along with the three highly influenced pathways as initial metabolic remodelling events that eventually evolve into the advanced stage cardiac hypertrophy and cardiac failure. Their application can be a screening tool for the onset of CH which would allow clinical intervention as early as possible. Thus, this study highlights the clinical implication of NAC that can be used to develop an animal model of CH and this model would help in future studies to overcome current limitations, especially in investigating the expression of Nrf2 along with the levels of glutathione which would indicate reductive stress.

## Author contributions

Both authors have equal contribution. 

Supplementary Materials
