# Supplementary material for: Identification of serum predictors of n-acetyl-l-cysteine and isoproterenol induced remodelling in cardiac hypertrophy
Source: Turk J Biol. 2021 Jun 23;45(3):323–32. doi: 10.3906/biy-2101-56 (PMC8313937; doi:10.3906/biy-2101-56)
Supplement: Supplementary file 1 — Supplementary Materials [file turkjbio-45-323-sup001.pdf]

Supplementary material

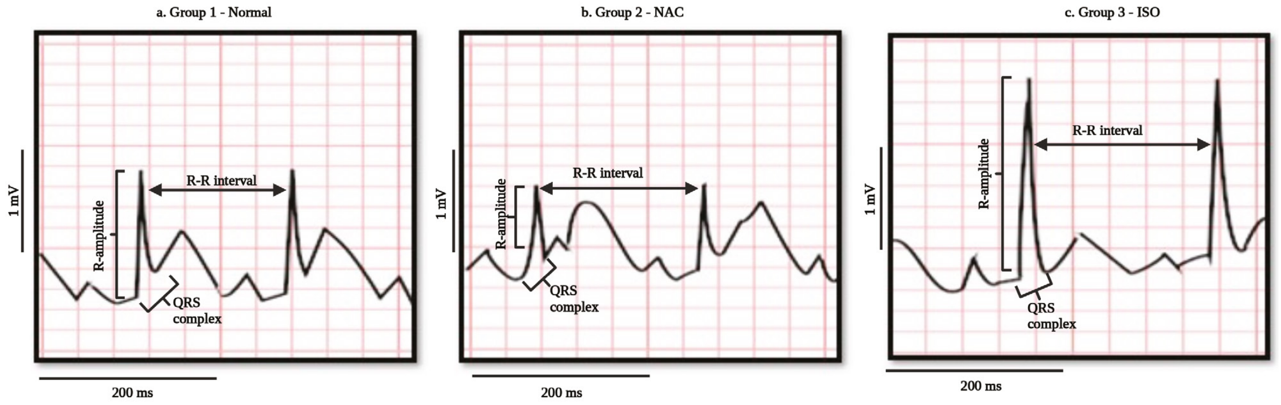

Figure S1. Graphical representation of CH using ECG (scaled to the real time raw ECG tracings). Typical CH characteristics namely widened QRS complex, elevated R-amplitude with prolonged R-R interval indicating delayed HR were observed in the standard model, ISO. Interestingly, NAC displayed widened QRS complex with impaired R-R interval and HR but with contrarily shortened R amplitude suggestive of the adverse fibrosis and an extended stage of CH, the dilated cardiomyopathy.

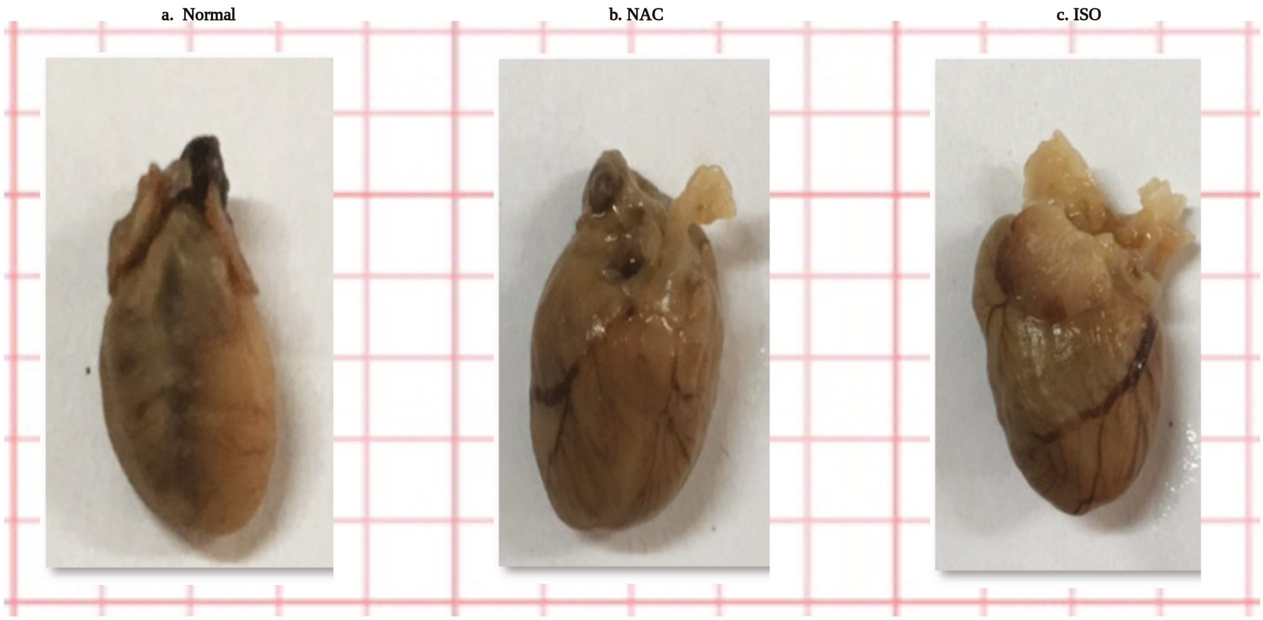

Figure S2. Morphological differences in heart sizes. When compared normal, the NAC and ISO administered rats revealed enlarged heart sizes in particular, the ventricles along with impaired arterial structures as shown in the figure that is scaled to graph.

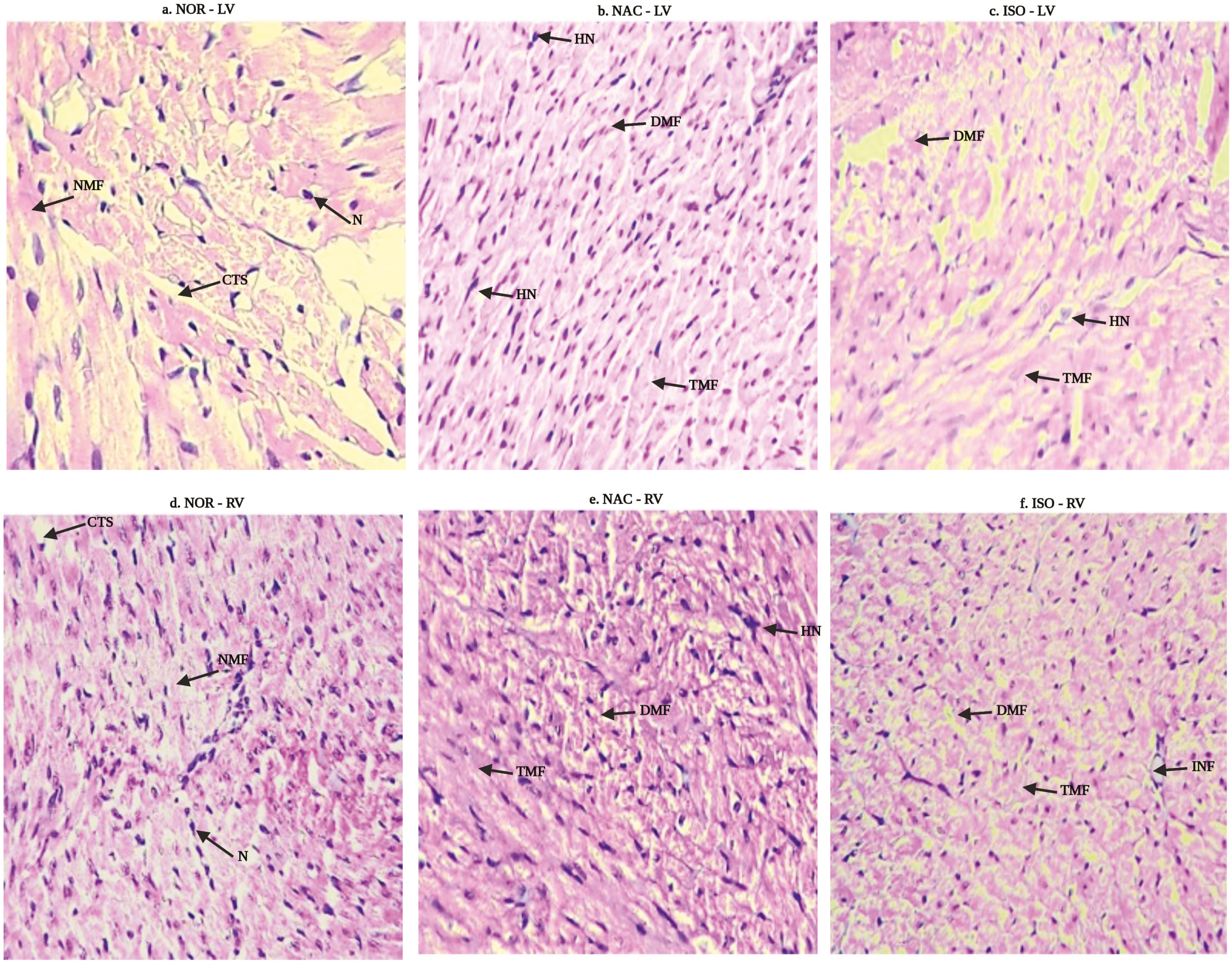

Figure S3. H&E staining of ventricles. (i) Left ventricle (LV): NAC (Figure b) and ISO (Figure c) displayed hypertrophied nuclei (HN) with distorted muscle fibers (DMF) and loss of connective tissue separations (CTS) when compared to normal (Figure a). (ii) Right ventricle (RV): similar patterns identified in LV were also observed in RV (Figures d to f). NAC revealed extensive thickened myofibrils (TMF) than ISO when compared to normal.

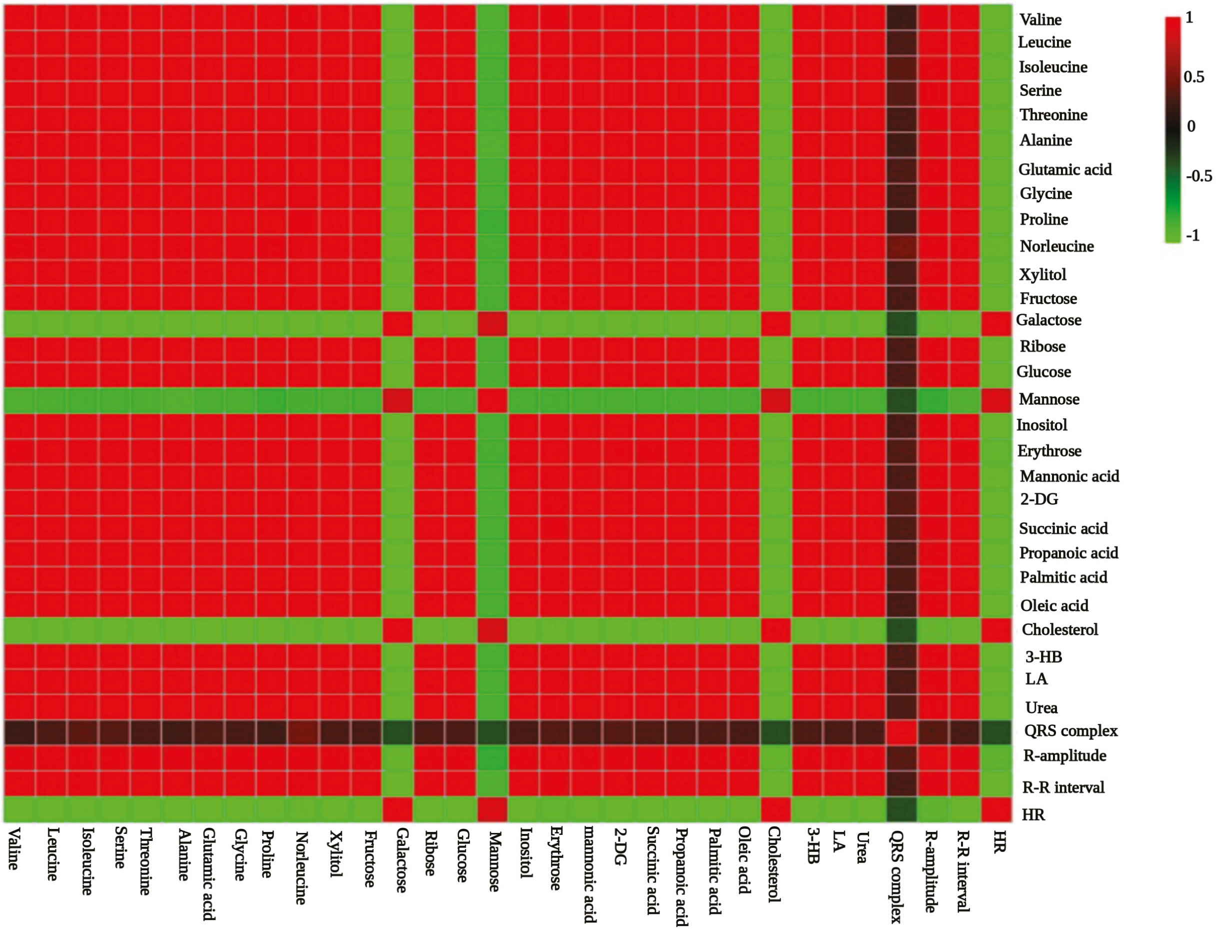

Figure S4. Correlation heat map for ISO induced CH. Unlike NAC, the ISO model revealed no strong correlating metabolites for QRS complex accompanied by metabolites that strongly correlated between themselves and other six metabolic responders among which mannose was found as least.

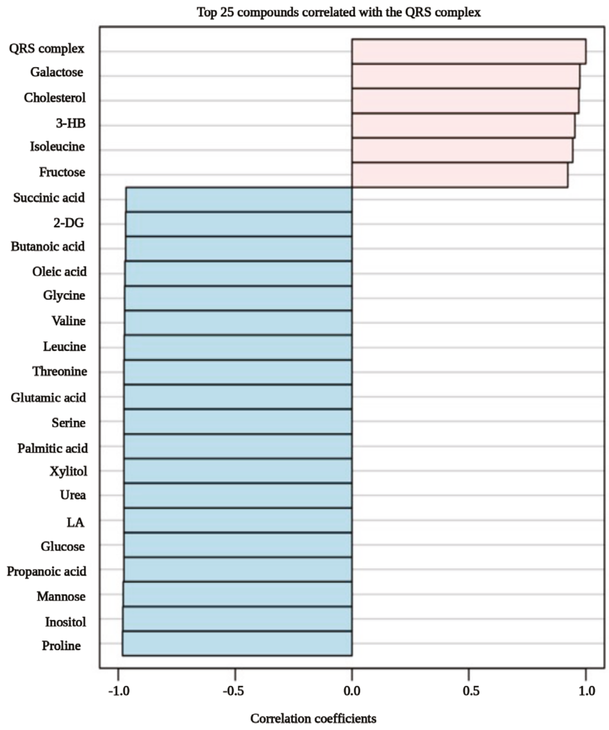

Figure S5a. Pattern hunting analysis to identify metabolites that correlate with QRS complex in NAC administered rat group. Metabolites that strongly correlate (coefficients > 0.7; positively correlated – pink; negatively correlated – blue) with the metabolite responder QRS complex are indicated.

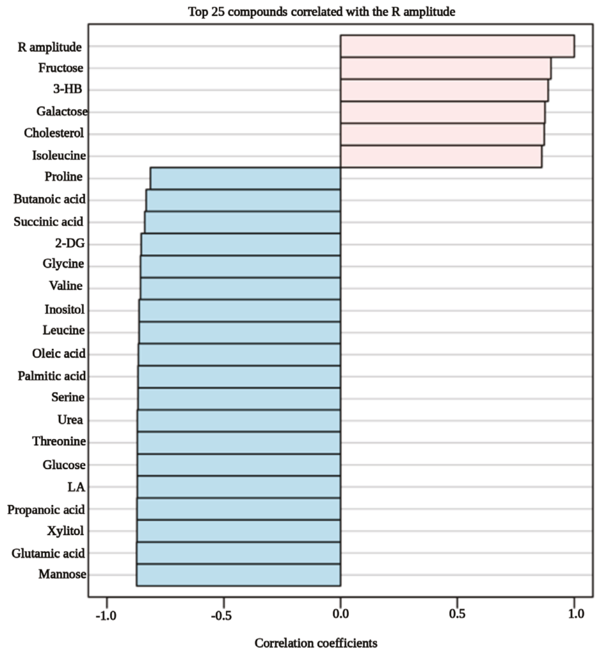

Figure S5b. Pattern hunting analysis to identify metabolites that correlate with R-amplitude in NAC administered rat group. Metabolites that strongly correlate (coefficients > 0.7; positively correlated – pink; negatively correlated – blue) with the metabolite responder R-amplitude are indicated.

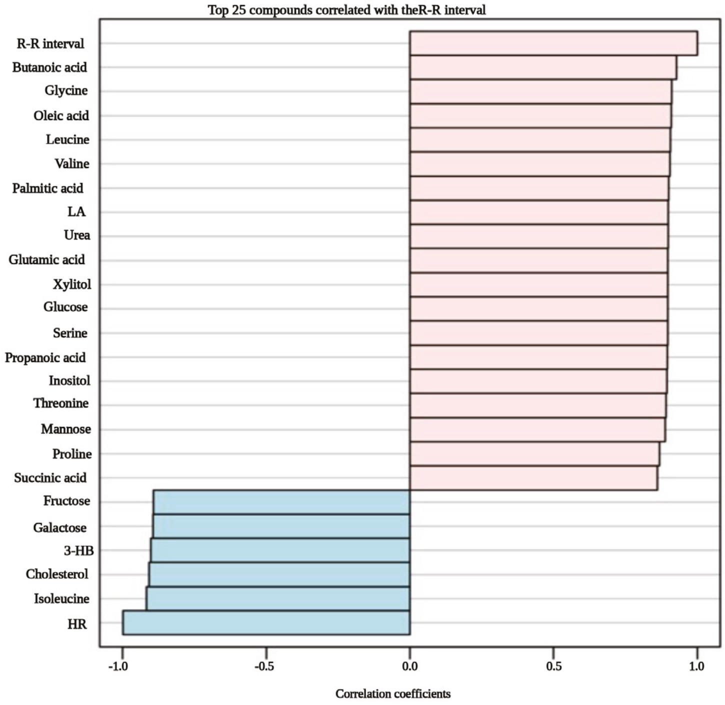

Figure S5c. Pattern hunting analysis to identify metabolites that correlate with R-R interval in NAC administered rat group. Metabolites that strongly correlate (coefficients > 0.7; positively correlated – pink; negatively correlated – blue) with the metabolite responder R-R interval are indicated.

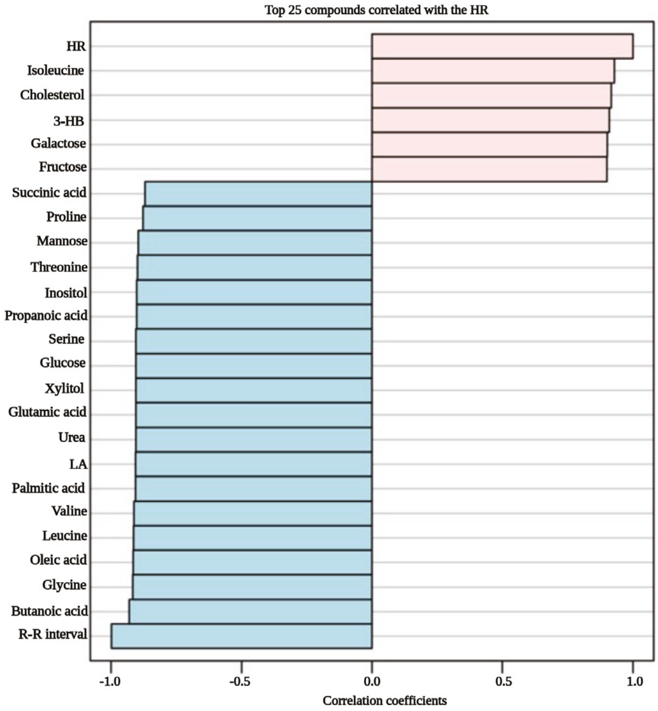

Figure S5d. Pattern hunting analysis to identify metabolites that correlate with heart rate in NAC administered rat group. Metabolites that strongly correlate (coefficients > 0.7; positively correlated – pink; negatively correlated – blue) with the metabolite responder heart rate are indicated.

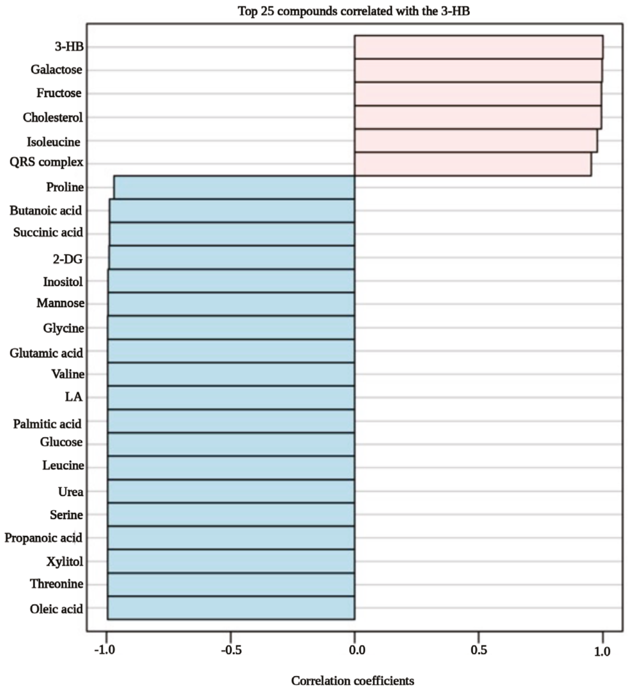

Figure S5e. Pattern hunting analysis to identify metabolites that correlate with the ketone body, 3-hydroxybutyrate (3-HB) in NAC administered rat group. Metabolites that strongly correlate (coefficients > 0.7; positively correlated – pink; negatively correlated – blue) with the metabolite responder 3-HB are indicated.

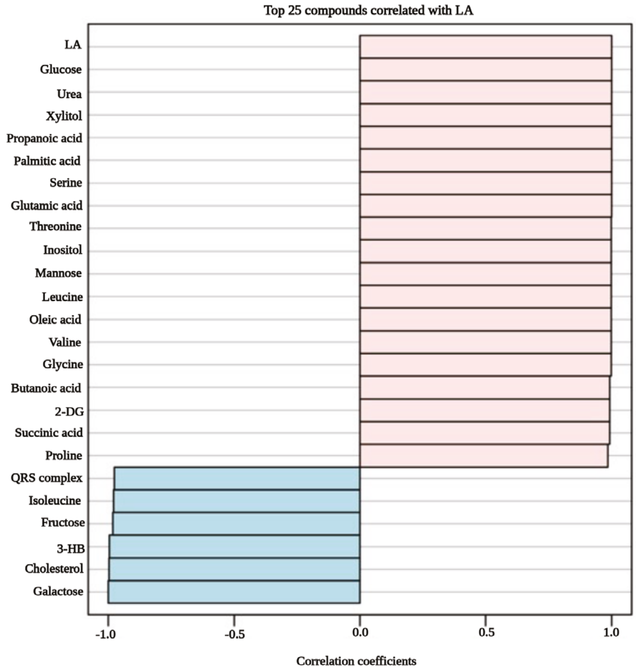

Figure S5f. Pattern hunting analysis to identify metabolites that correlate with lactic acid (LA) in NAC administered rat group. Metabolites that strongly correlate (coefficients > 0.7; positively correlated – pink; negatively correlated – blue) with the metabolite responder LA are indicated.

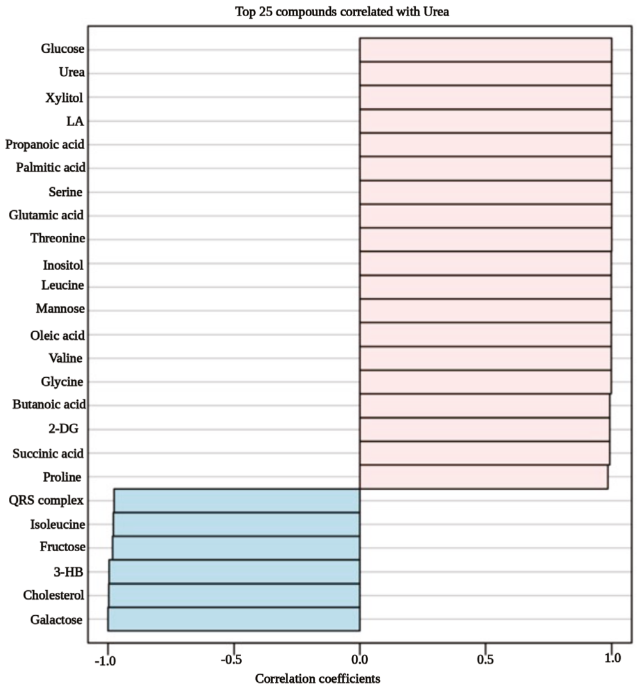

Figure S5g. Pattern hunting analysis to identify metabolites that correlate with urea in NAC administered rat group**.** Metabolites that strongly correlate (coefficients > 0.7; positively correlated – pink; negatively correlated – blue) with the metabolite responder urea are indicated.

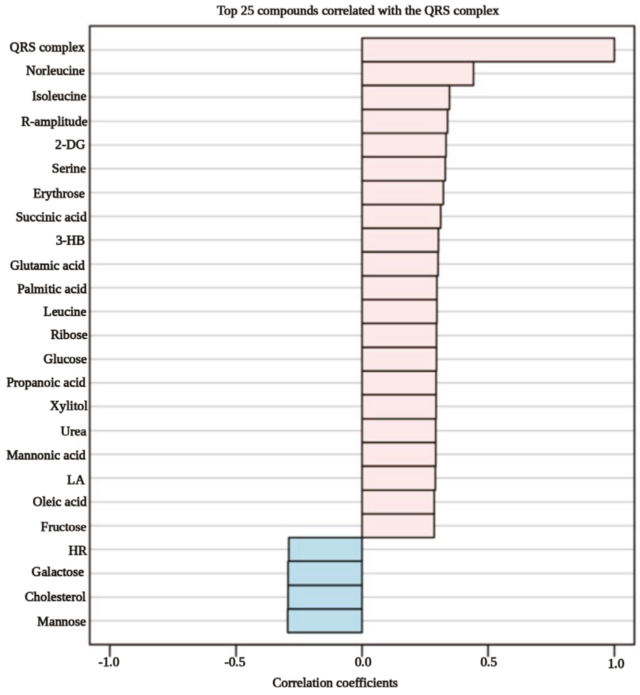

Figure S6a. Pattern hunting analysis to identify metabolites that correlate with QRS complex in ISO administered rat group. Metabolites that strongly correlate (coefficients > 0.7; positively correlated – pink; negatively correlated – blue) with the metabolite responder QRS complex are indicated.

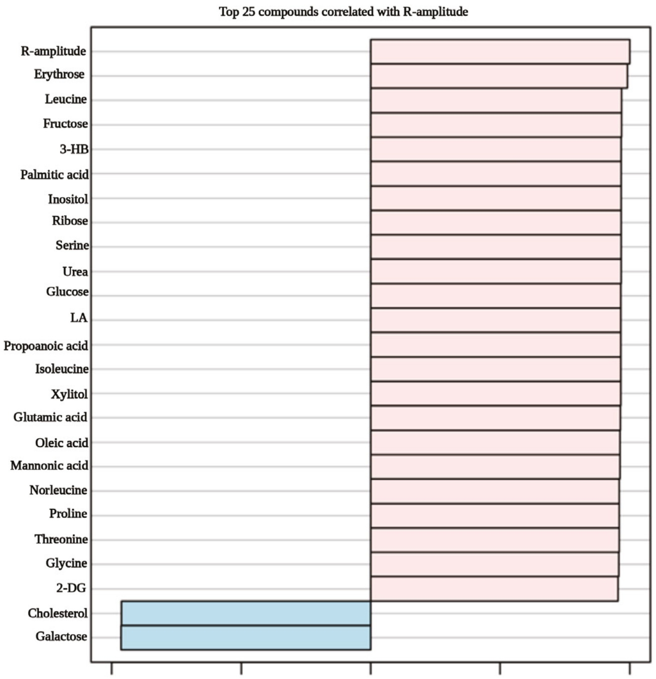

Figure S6b. Pattern hunting analysis to identify metabolites that correlate with R-amplitude in ISO administered rat group. Metabolites that strongly correlate (coefficients > 0.7; positively correlated – pink; negatively correlated – blue) with the metabolite responder R-amplitude are indicated.

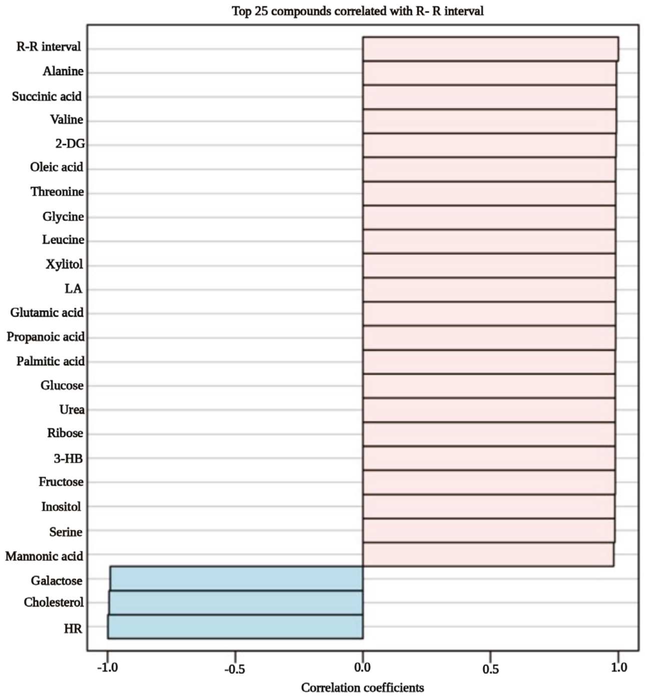

Figure S6c. Pattern hunting analysis to identify metabolites that correlate with R-R interval in ISO administered rat group. Metabolites that strongly correlate (coefficients > 0.7; positively correlated – pink; negatively correlated – blue) with the metabolite responder R-R amplitude are indicated.

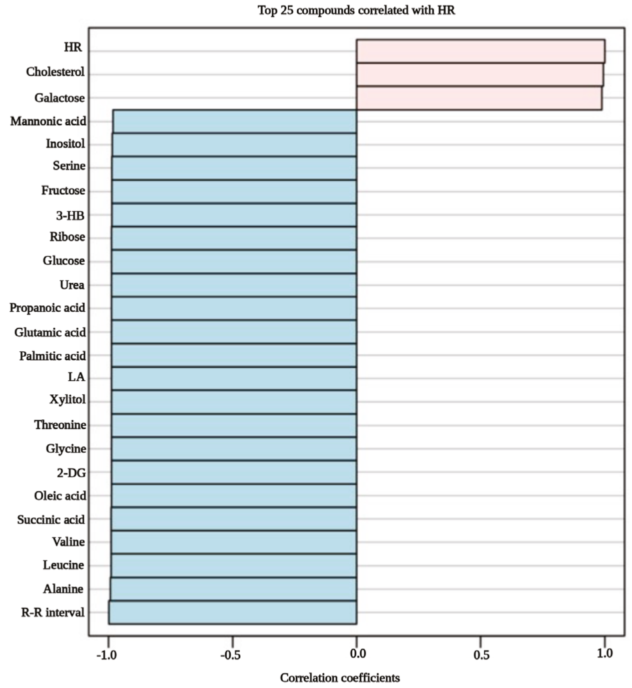

Figure S6d. Pattern hunting analysis to identify metabolites that correlate with heart rate (HR) in ISO administered rat group. Metabolites that strongly correlate (coefficients > 0.7; positively correlated – pink; negatively correlated – blue) with the metabolite responder HR are indicated.

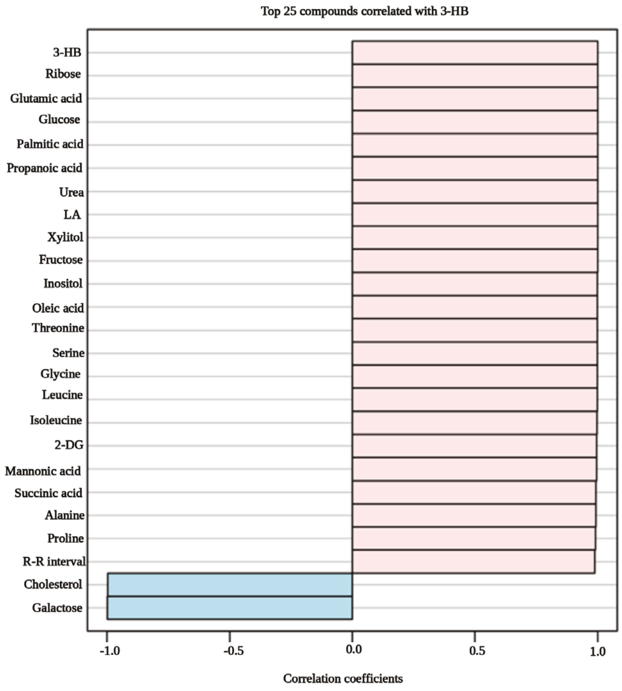

Figure S6e. Pattern hunting analysis to identify metabolites that correlate with ketone body, 3-hydroxybuytrate (3-HB) in ISO administered rat group. Metabolites that strongly correlate (coefficients > 0.7; positively correlated – pink; negatively correlated – blue) with the metabolite responder 3-HB are indicated.

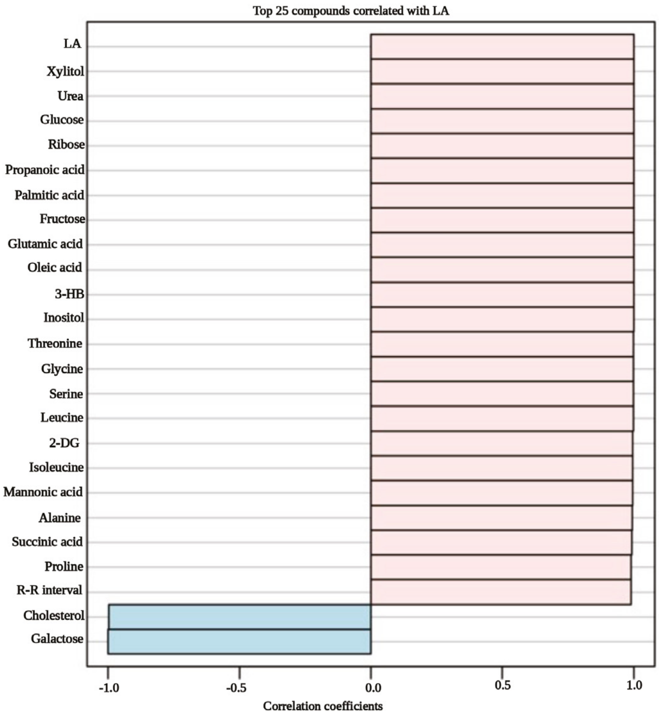

Figure S6f. Pattern hunting analysis to identify metabolites that correlate with ketone body, 3-hydroxybuytrate (3-HB) in ISO administered rat group**.** Metabolites that strongly correlate (coefficients > 0.7; positively correlated – pink; negatively correlated – blue) with the metabolite responder 3-HB are indicated.

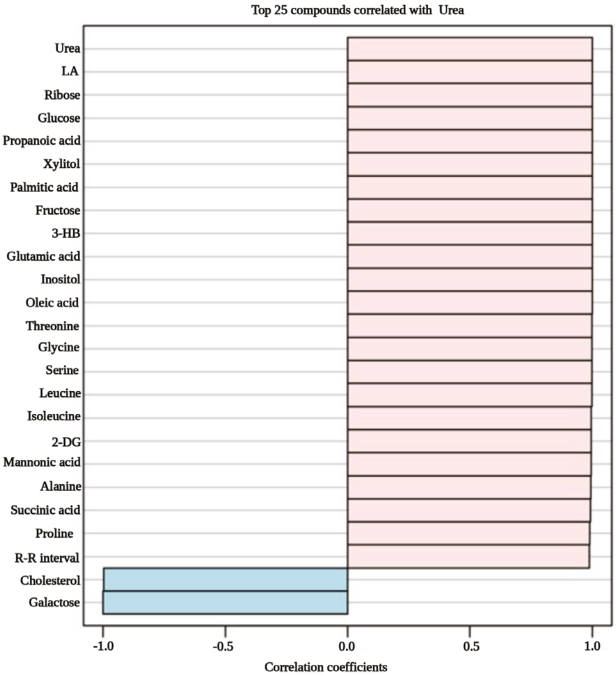

Figure S6g. Pattern hunting analysis to identify metabolites that correlate with urea in ISO administered rat group. Metabolites that strongly correlate (coefficients > 0.7; positively correlated – pink; negatively correlated – blue) with the metabolite responder urea are indicated.

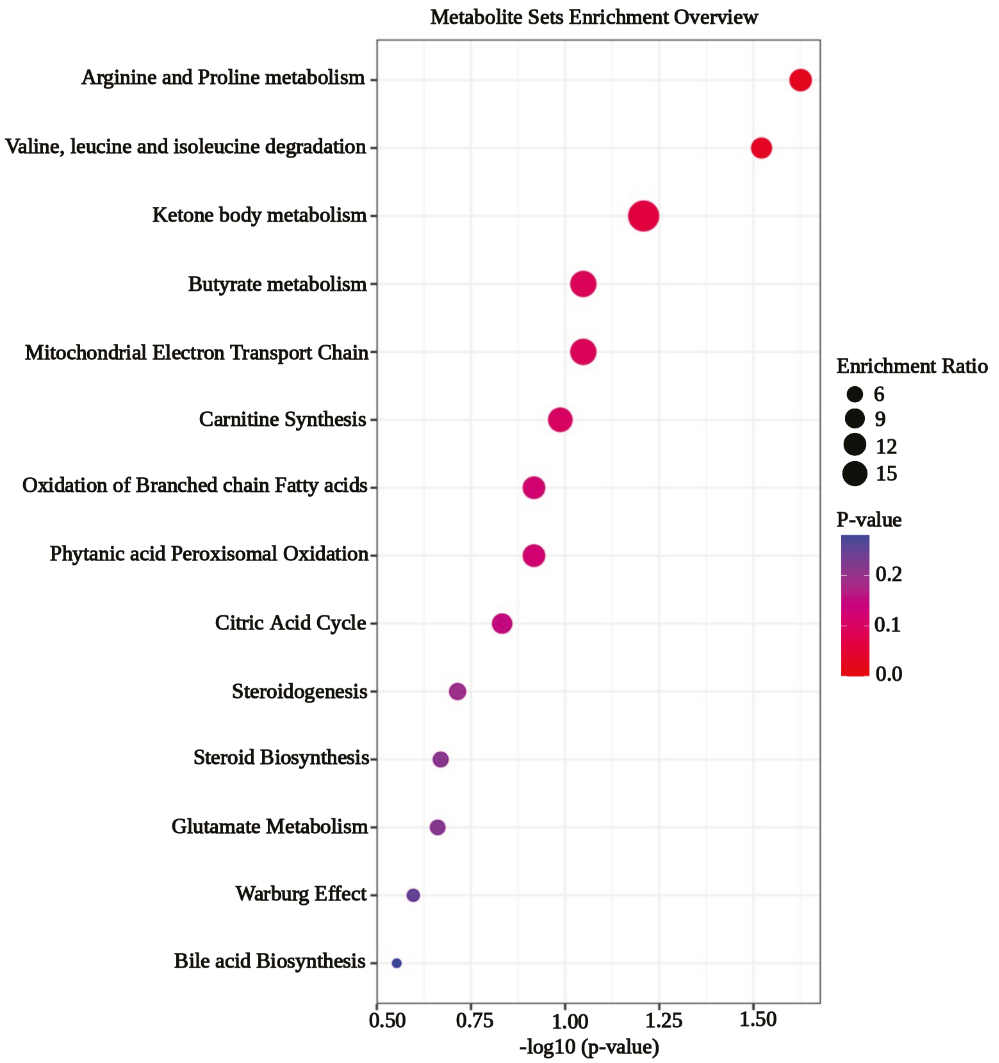

Figure S7. Pathway mapping by MSEA in ISO model. Arginine and proline metabolism along with BCAA degradation are commonly shown as significantly influenced in both CH models ($p < 0.05$). Though there are other highly impacted metabolic pathways listed in the figure, their level of significance, $p > 0.05$.
